# MiR-650 represses high-risk non-metastatic colorectal cancer progression via inhibition of AKT2/GSK3β/E-cadherin pathway

**DOI:** 10.18632/oncotarget.17743

**Published:** 2017-05-10

**Authors:** Chunxian Zhou, Fengyun Cui, Jiali Li, Diyi Wang, Yingze Wei, Ying Wu, Jiping Wang, Hongguang Zhu, Shuyang Wang

**Affiliations:** ^1^ Department of Pathology, School of Basic Medical Sciences, Fudan University, Shanghai, China; ^2^ Department of Pathology, Shanghai University of Traditional Chinese Medicine, Shanghai, China; ^3^ Division of Surgical Oncology, Brigham and Women Medicine, Harvard Medical School, Boston, Massachusetts, USA

**Keywords:** non-metastatic colorectal cancer, prognosis biomarker, microRNA, AKT pathway

## Abstract

Although 5-year survival rate of non-metastatic colorectal cancer (CRC) is high, about 10% of patients in stage I and II still develop into metastatic CRC and eventually die after resection. Currently, there is no effective biomarker for predicting the prognosis of non-metastatic CRC in clinical practice. In this study, we identified miR-650 as a biomarker for prognosis prediction. We observed that the expression of miR-650 in tumor tissues had a positive association with overall survival. MiR-650 inhibited cell growth and invasion *in vitro* and *in vivo*. Furthermore, miR-650 targeted AKT2 and repressed the activation of the AKT pathway (AKT2/GSK3β/E-cadherin). Thus it induced the translocation of E-cadherin and β-catenin in cancer cells. Our results highlight the potential of miR-650 as a prognostic prediction biomarker and therapeutic target in non-metastatic CRC via inhibition of the AKT2/GSK3β/E-cadherin pathway.

## INTRODUCTION

Colorectal cancer (CRC) is one of the leading causes of cancer mortality worldwide. About 10 percent of CRC patients in stage I and II develop tumor metastasis and eventually die within 5 years after resection [1]. Therefore, identifying high-risk early-stage (stage I and II) CRC patients for aggressive chemotherapy is critical to improving patient outcome and avoiding unnecessary chemotoxicity. The current tools to assess CRC prognosis include the AJCC TNM staging system, other clinical and pathological variables such as obstruction, perforation, lymph vascular invasion, aneuploidy and molecular markers such as loss of heterozygosity (LOH) of 18q or the presence of microsatellite stable tumors, K-ras mutation, stromal matrix metalloproteinase-2 expression, group IIA phospholipase A2, 5q retention and tumor stroma ratio [2–8]. The clinical significance of these markers is incompletely understood, and many are either limited to a small portion of the patient population or exhibit unsatisfactory assay performance. Therefore, it is critical to either identify newer predictive and prognostic markers or to discover new target for treatment of high-risk early-stage CRC patients.

MicroRNAs (miRNAs) are small noncoding RNAs which repress gene expressions by recognizing complementary target sites in the 3′-UTRs of mRNAs [9]. MiRNAs, having strong associations with clinical outcomes of cancer [10, 11], have become major potential prognostic biomarkers for cancer patients. For example, miR-320 and miR-498 are identified to be predictive biomarkers for high-risk stage-II colon cancer patients [12]. MiR-21 expression appears to identify high-risk stage-II colon cancer patients as well [13–16]. However, miR-21 can be detected in both tumor cells and tumor stroma cells, and its function in CRC remains unclear. Therefore, it is still questionable whether miR-21 qualifies as a therapy target. Although some studies on miRNA expression of CRC prognosis have been reported [17–19], the majority of these studies used the whole tumor tissues as study material. Analyses of such complex tissues could conceal the specific signature of the truly transformed cancerous cells [20]. In order to discover reliable biomarkers, we macrodissected the tumor tissues and made sure that at least 75 percent of the samples were cancer cells.

MiR-650 plays different roles in several types of cancer. Mraz *et al*. have reported that higher expression of miR-650 is associated with a favorable prognosis in chronic lymphocytic leukemia (CLL) [21]. On the other hand, some reports have shown that miR-650 could stimulate metastasis in gastric cancer [22] and promote hepatocellular carcinoma [23]. The inconsistent reports suggest that miR-650 plays different roles in different cancers or in the different stages of a single cancer. Although the expression correlation between miR-650 and NDRG2 (a target gene) in CRC has been reported, the function and downstream pathway of miR-650 in CRC are still unclear [24]. Furthermore, the relationship between the expression profile of miR-650 and prognosis of non-metastatic CRC is also unclear.

In this study, we discovered that the expression of miR-650 in CRC tumor tissues had a strong positive association with overall survival (OS) of non-metastatic CRC patients. Our data indicated that miR-650 inhibited CRC cells *in vitro* and *in vivo* by repressing the AKT2 pathway. MiR-650 had considerable clinical value in the prognosis prediction of non-metastatic CRC and potential target therapy in early-stage CRC.

## RESULTS

### MiR-650 expression has a positive association with the prognosis of early-stage CRC

**Figure 1:**
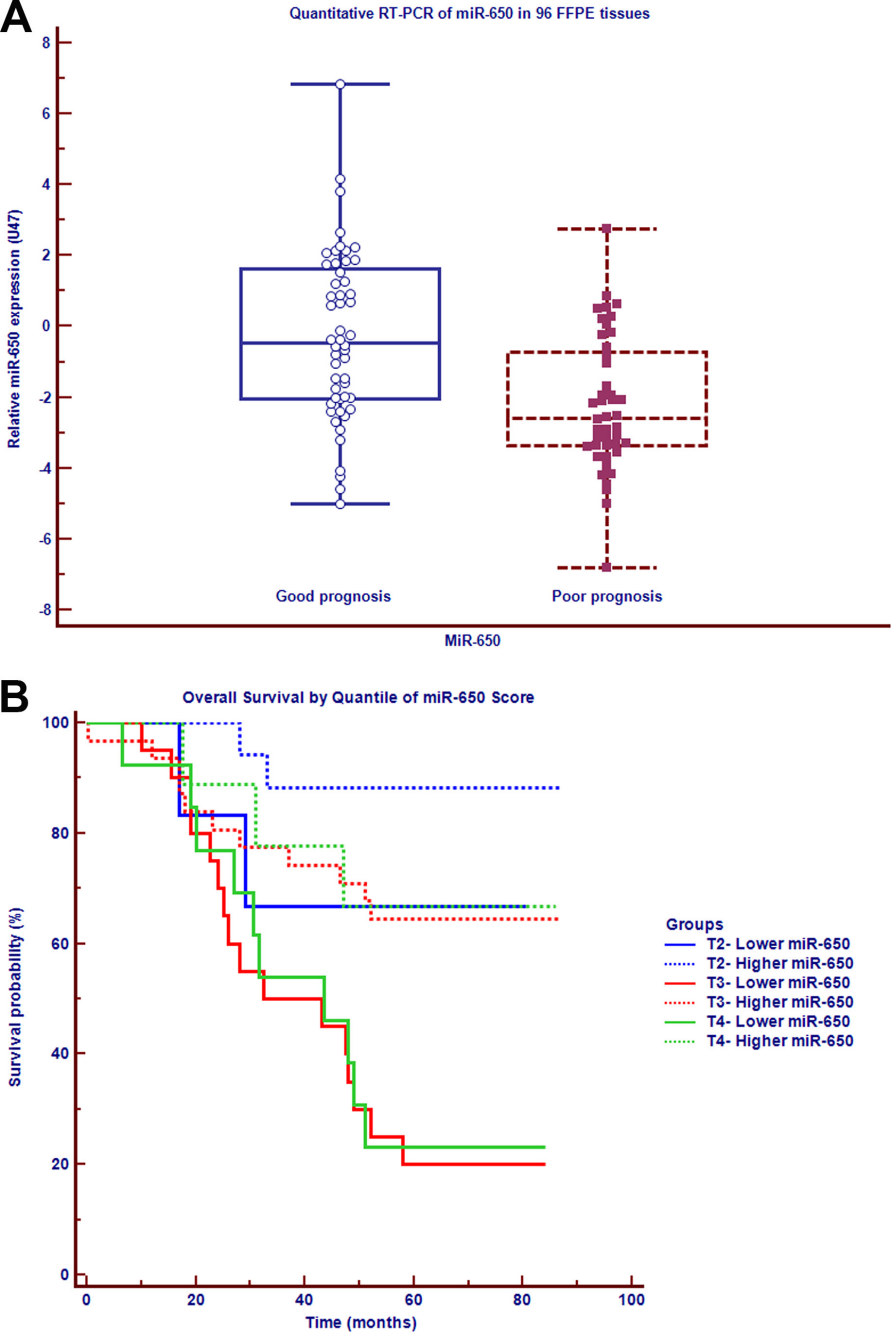
MiR-650 is positively associated with the prognosis of early-stage CRC (**A**) MiR-650 expression in carcinoma cells among 96 non-metastasis CRC patients with different prognosis on qRT-PCR. (**B**) Survival analysis in different tumor stages of non-metastasis CRC patients.

The clinical, histopathological, and survival details of the 96 cases were summarized in Table 1. MiRNA microarray was used to measure the expression of human miRNAs in tumor cells derived from 8 formalin-fixed, paraffin-embedded (FFPE) samples among early-stage CRC patients. Comparison of patients with poor prognosis to those with good prognosis revealed three under-expressed miRNAs (Fold change < 0.5, *P* <= 0.05) and two over-expressed miRNAs (Fold change > 2, *P* < 0.05) (GEO accession number GSE79810, Supplementary Table 1). Among those miRNAs, miR-650 was under-expressed (Fold change 0.3, *P* = 0.05). This result was further validated using reverse-transcriptase-polymerase-chain-reaction (qRT-PCR) analysis on a total of 96 early-stage CRC samples. MiR-650 was significantly under-expressed in 44 patients with poor prognosis as compared with 52 patients with good prognosis (Fold change 0.13, *P* < 0.01) (Figure 1A). From the ROC analysis, miR-650 expression <= 0.2382 was considered relatively lower expression and > 0.2382 was considered relatively higher expression (AUC = 0.745). To further clarify the association between miR-650 expression and OS with different stages of tumor pathology in non-metastatic CRC, we performed survival analysis. As shown in Figure 1B, patients with a relatively lower expression of miR-650 had shorter OS than those with a relatively higher expression, even at different T stages (T2, T3 and T4): significantly positive associations were detected at T3 and T4 (T3: hazard ratio [HR] = 6.68, 95% CI: 2.55–17.52, *P* < 0.01; T4: HR = 3.48, 95% CI: 1.06–11.44, *P* < 0.05), respectively. The similar association pattern was observed at T2 (HR = 4.54, 95% CI: 0.44–46.48, *P* > 0.05). We conclude that miR-650 has the potential to be a prognostic predictive biomarker in non-metastatic CRC.

**Table 1:** Characteristics of patients and tumors in the study

	Microarrays		qRT-PCR (including samples from discovery and validation phases)	
Characteristic	Good Prognosis^a^	Poor Prognosis^b^	Good Prognosis^a^	Poor Prognosis^b^
**Number of Cases**	4	4	52	44
**Gender**				
Male	2	1	23	19
Female	2	3	29	25
**Age at Diagnosis**				
Median	67	73	67	70
Min	57	67	47	50
Max	78	81	89	91
**Survival Time (months)**				
Median	72.3	28.8	75.9	30.7
Min	61	17	60	0.2
Max	84	51	87	58
**TNM Stage of Diseases^c^**				
Tumour (T) Stage				
T1	0	0	0	0
T2	1	0	19	4
T3	3	4	24	27
T4	0	0	9	13
Nodal (N) Status	0	0	0	0
Distant Metastases(M)	0	0	0	0
**Location of CRC**				
Left	1	1	3	5
Right	2	1	16	12
Rectum and Sigmoid	1	2	31	23
Transverse	0	0	1	3
Unknown	0	0	1	1

^a^Patients with good prognosis were all alive more than 5 years after surgery.

^b^Patients with poor prognosis were all died of CRC recurrence within 5 years after surgery.

^c^All the patients observed had no metastasis at the time of diagnosis and did not have chemotherapy.

### MiR-650 inhibits cell proliferation *in vitro* and *in vivo*

**Figure 2:**
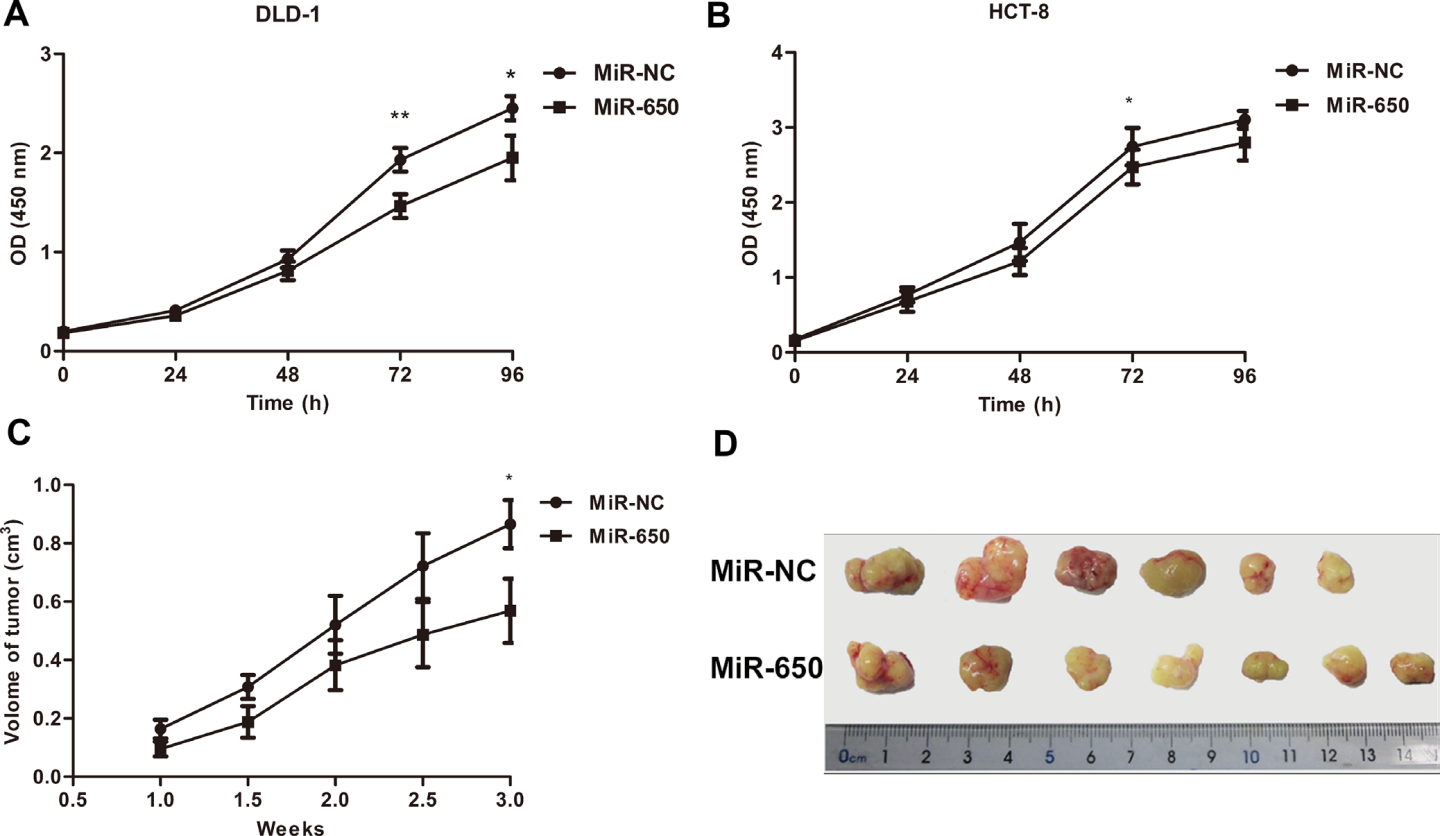
MiR-650 inhibits cell growth *in vitro* and *in vivo* (**A**) MiR-650 inhibited cell growth on 72 h and 96 h in DLD-1 (**P = 0.07, *P < 0.05). (**B**) MiR-650 significantly inhibited cell growth on 72 h in HCT-8 (*P < 0.05). (**C**) MiR-650 had a growth inhibitory effect *in vivo* during 3 weeks in subcutaneous tumor mice model (*P = 0.05). (**D**) In subcutaneous tumor mice model, the volume of tumors in NC group was larger than that of tumors in miR-650 group.

The significant under-expression of miR-650 in the poor prognosis group led us to explore the biological functions of miR-650 in colon cancer cells. We also found expression of miR-650 was significantly decreased in carcinomas compared to normal tissues in our previous study [25]. We tested the basal expression levels of miR-650 in seven colon cancer cell lines (Supplementary Figure 1A). DLD-1 cells showed a relatively lower expression level of miR-650 compared with other cells, while HCT-8 showed a moderate expression level of miR-650. The miR-650 precursor was stably transfected to DLD-1 and HCT-8 cells with lentiviral vectors, and miR-650 expression was increased 5.0 fold in DLD-1 transfectants and 3.2 fold in HCT-8 transfectants compared with NC controls (transfected with scrambled control sequence) (Supplementary Figure 1B). As illustrated in Figure 2, miR-650 significantly inhibited cell growth in both DLD-1 (21.0 ± 9.7% reduction at 96 h, *P* < 0.05, Figure 2A) and HCT-8 cell lines (9.9 ± 2.4% reduction at 72 h, *P* < 0.05, Figure 2B). These data indicated a growth inhibitory role of miR-650 *in vitro*. *In vivo* experiment with DLD-1 cell line in the subcutaneous tumor mice model showed that the tumor volumes were 0.87 ± 0.22 cm^3^ in the NC control and 0.57 ± 0.29 cm^3^ in the miR-650 transfectant group, respectively (*P* = 0.05, Figure 2C, 2D). Meanwhile, Ki-67 expression in miR-650 group showed a lower score compared with the NC group in tumor xenograft mice (Fold change 0.64, *P* = 0.09, Supplementary Figure 2). These data indicated that miR-650 could inhibit tumor growth *in vivo*.

### MiR-650 inhibits cell migration and invasion *in vitro* and *in vivo*

**Figure 3:**
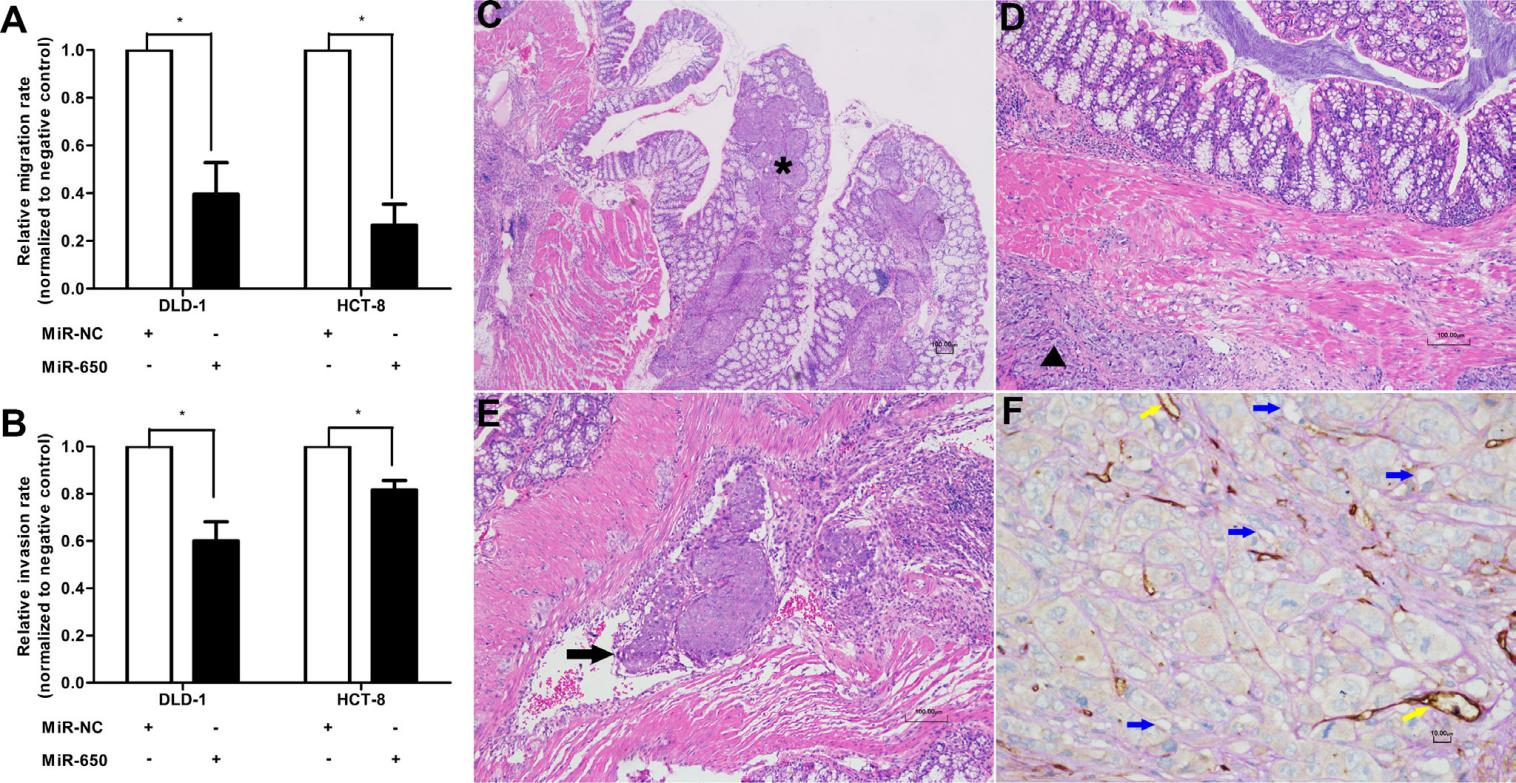
MiR-650 inhibits cell migratory and invasive abilities (**A**) Cell migratory abilities in DLD-1 and HCT-8 cells. *P < 0.05 vs. NC group. (**B**) Cell invasive abilities in DLD-1 and HCT-8 cells. *P < 0.05 vs. NC group. (**C**) Representative figure of NC group. Cancer cells invaded from colon serous to epithelial tissue (⋆HE 100×). (**D**) Representative figure of miR-650 group. Cancer cells invaded from colon serous to submucosa tissue (▲HE 100×). (**E**) Representative figure of tumor cell embolus in xenograft mice model (thick arrow, HE 100×). (**F**) Representative VM channels in xenograft mice model. The endothelium-independent vessels (VM) (blue arrows) were lined by tumor cells, and PAS-positive substances enclosed the tumor cells to form the basement membrane-like structure. However, the endothelium marker CD34 was negative. The endothelium-dependent vessels (yellow arrows) were CD34 positive, and PAS positive (400×).

The potential impact of miR-650 on cell migration and invasion were assessed by transwell assays. MiR-650 transfectants had significantly reduced migration and invasion rates compared with NC controls in both DLD-1 and HCT-8 cells (Figure 3A, 3B). The migratory abilities were inhibited by miR-650 compared with NC controls in DLD-1 cells (60.4 ± 23.0% reduction, *P* < 0.05) and in HCT-8 cells (73.4 ± 15.3% reduction, *P* < 0.05) (Figure 3A, Supplementary Figure 3A). The invasive abilities were inhibited by miR-650 compared with NC controls in DLD-1 cells (60.0 ± 14.2% reduction, *P* < 0.05) and in HCT-8 cells (81.6 ± 6.9% reduction, *P* < 0.05) (Figure 3B, Supplementary Figure 3B).

We planted tumor tissues from the two groups of subcutaneous tumor models on colon serosa of tumor xenograft mice. We observed that cancer cells invaded from serous to colon epithelial tissue in the animals from the NC group (Figure 3C), while cancer cells only invaded from serous to colon submucosa tissue in the animals from the miR-650 group (Figure 3D). Meanwhile, tumor thrombi were found in 6 out of 8 mice in the NC group but were only found in 3 out of 7 mice in the miR-650 group (Figure 3E). Furthermore, according to the VM identification in the xenograft model, fewer VM channels (1.27 ± 0.30) were found in the miR-650 group than in the NC group (1.63 ± 0.21) (*P* < 0.05) (Figure 3F, Table 2, Supplementary Figure 4). Therefore, we confirmed that miR-650 might inhibit the tumor invasion by blocking the blood supply of tumor cells in CRC.

**Table 2:** Number of VM channels in xenograft mice model

Mice model No.	VM number^a^							Mean ± SD	*P*. Value
	1	2	3	4	5	6	7		
MiR-NC	1.6	1.8	1.2	1.8	1.8	1.6	1.6	1.63 ± 0.21	0.026
**MiR-650**	1.2	1.2	1.2	1.6	1.5	0.7	1.5	1.27 ± 0.30	

^a^Mean of every ten 400× fields per mouse model.

### MiR-650 targets to AKT2 and represses AKT pathway

To understand the underlying mechanisms that are implicated in the impact of miR-650 on the prognosis of early-stage CRC, potential target genes were searched using miRecords and examined using the KEGG database. *AKT2* was predicted as a candidate target gene of miR-650.

**Figure 4:**
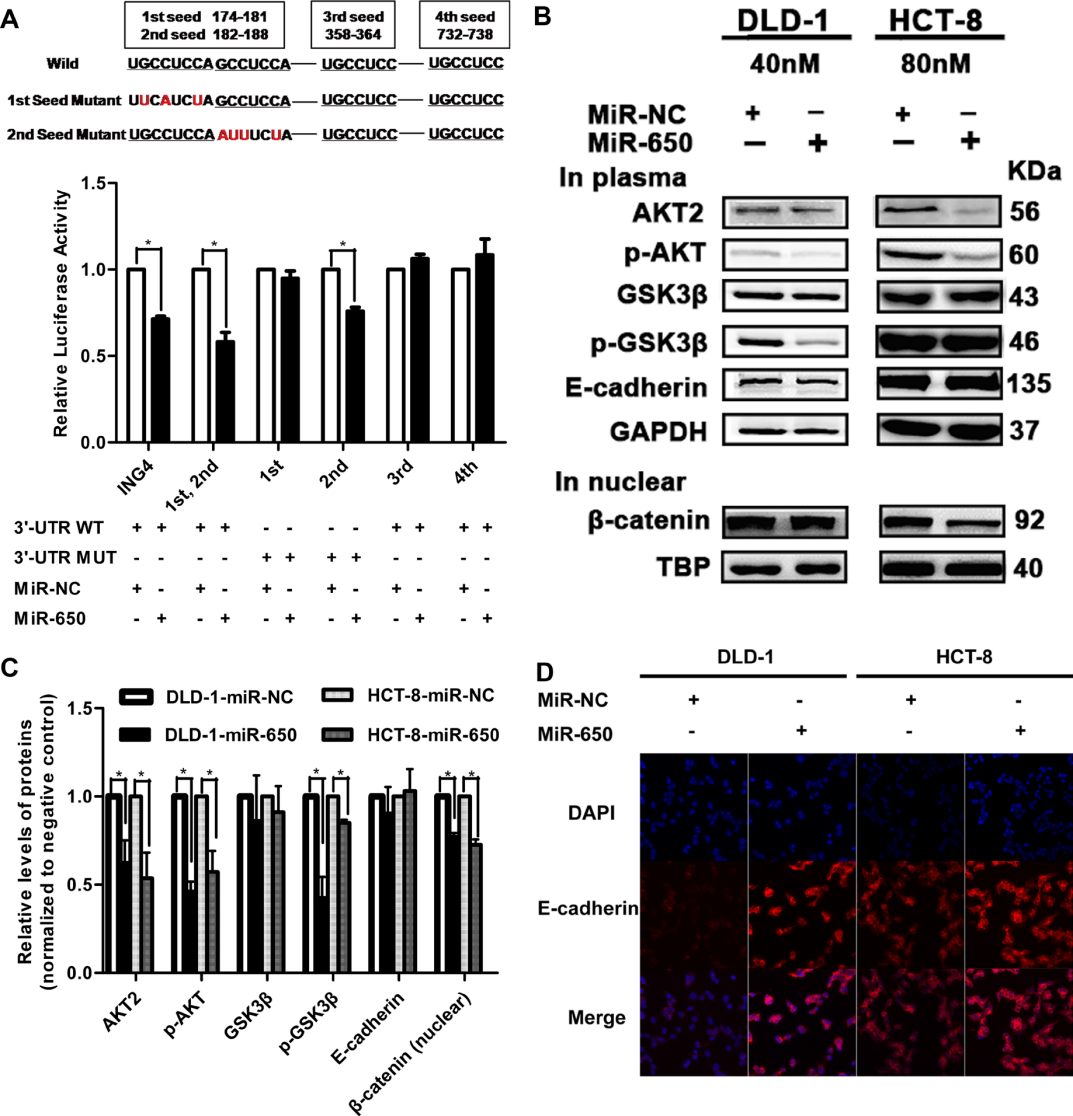
MiR-650 targets to *AKT2*. *P < 0.05 vs. NC control (**A**) Luciferase assays. *ING4* was used as a positive control. (**B**–**C**) Western blot examined AKT2, p-AKT, GSK3β, p-GSK3β, E-cadherin of total protein and β-catenin of nuclear protein. (**D**) Immunofluorescence of E-cadherin was increased on cell surface in DLD-1 cells and HCT-8 cells by miR-650 (400×).

*AKT2* has four seed sequences that may combine with miR-650 (Supplementary Table 2). To identify the miR-650 binding site on *AKT2*, we performed a series of luciferase assays. The luciferase activity of the vector with the combination of the 1st and 2nd seed sequence was significantly reduced to 58.1 ± 9.7% in cells transfected with miR-650 precursor compared with negative control cells (*P* < 0.05). There were no significant luciferase activity changes of vectors with the 3rd or 4th seeds (Figure 4A). In addition, the mutation experiment showed that the reduction of luciferase activities disappeared when the 1st seed sequence was mutated, but this phenomenon was not observed when the 2nd seed sequence was mutated (Figure 4A). This observation indicated that the binding site of miR-650 was located at the 1st seed.

Furthermore, the activation of AKT pathway was also investigated after the miR-650 precursor was transfected in DLD-1 and HCT-8 cells. As shown in Figure 4B and 4C, the protein levels of AKT2 were decreased by 37.3 ± 12.7% (*P* < 0.05) and 45.9 ± 14.6% (*P* < 0.05) in DLD-1 and HCT-8 cells, respectively. The expression levels of phosphorylated AKT (p-AKT, Ser473) were decreased by 53.6 ± 5.6% (*P* < 0.05) and 42.0 ± 12.1% (*P* < 0.05), respectively, while p-GSK3β (Ser9) levels were decreased by 58.4 ± 12.0% (*P* < 0.05) and 15.2 ± 1.9% (*P* < 0.05), respectively. Nevertheless, there were no significant changes in GSK3β expression levels after transfection in both two cell lines (*P* > 0.05, Figure 4B, 4C). E-cadherin is a main component of cell-cell adhesion junctions. Suppression or mislocalization of E-cadherin from the cell membrane was observed in CRC patients [26]. Interestingly, although the total expression level of E-cadherin had no significant difference after transfection of the miR-650 precursor in DLD-1 and HCT-8 cells (*P* > 0.05, Figure 4B, 4C), dramatically increased E-cadherin expression levels were observed on the cell surfaces in miR-650 stable transfectants (Figure 4D, Supplementary Figure 5). E-cadherin sequestered β-catenin to cell surfaces and the translocation of β-catenin to nucleus activated the Wnt/β-catenin pathway [27]. We observed that β-catenin on the cell membrane increased in DLD-1 miR-650 stable transfectants (Supplementary Figure 6). β-catenin levels in the nucleus were also decreased by 22.8 ± 2.1% (*P* < 0.05) and 27.5 ± 3.0% (*P* < 0.05) when transfected with miR-650 precursor in DLD-1 and HCT-8 cells (Figure 4B, 4C).

### AKT2 overexpression restores cell migratory/invasive abilities and proliferation inhibited by miR-650

**Figure 5:**
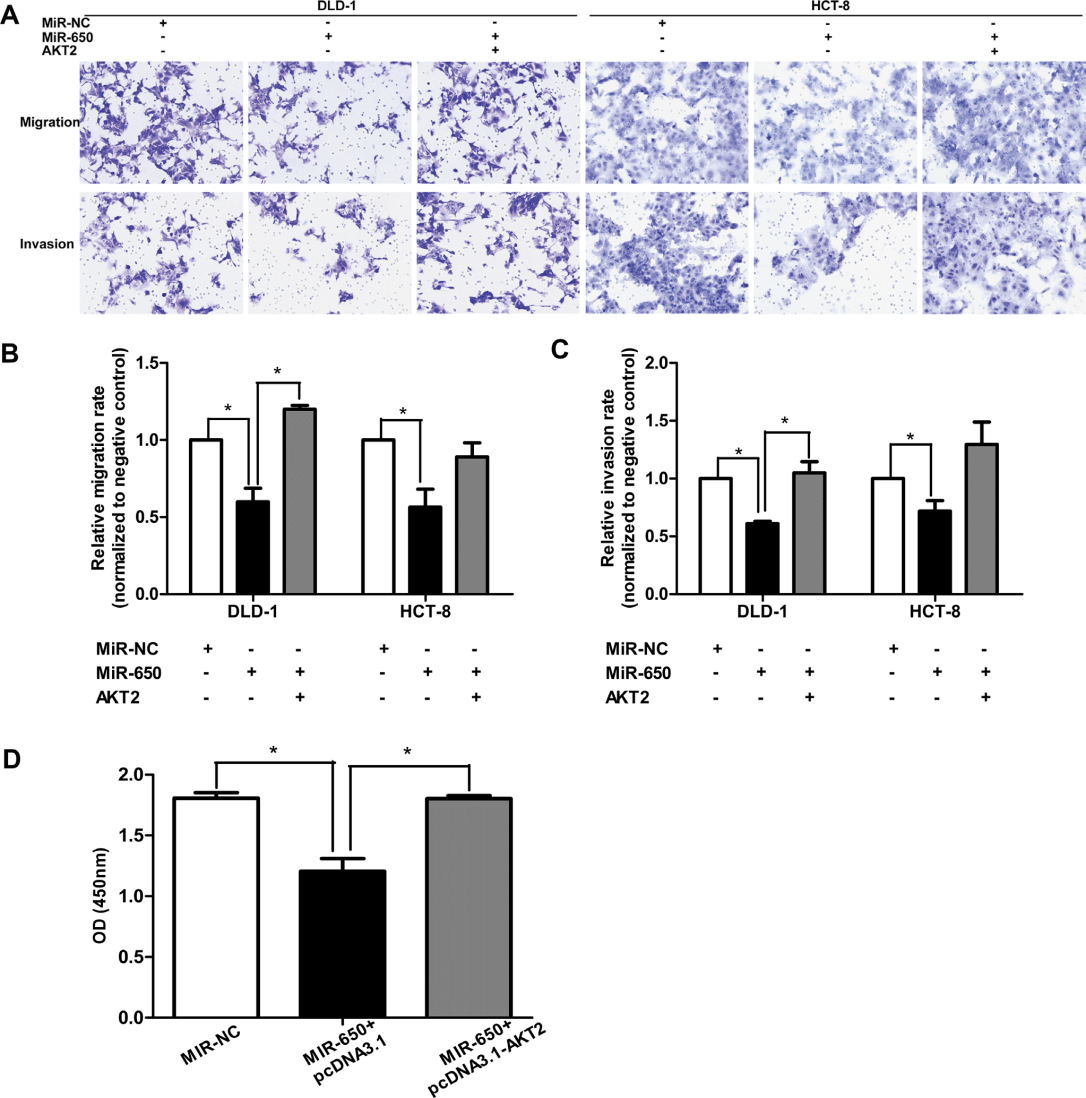
AKT2 overexpression restores cell migratory/invasive abilities and proliferation (**A**–**C**) AKT2 overexpression restored cell migratory and invasive abilities which were inhibited by miR-650 in DLD-1 and HCT-8 cells (200×). *P < 0.05. (**D**) AKT2 overexpression restored cell proliferation inhibited by miR-650 in DLD-1 cells. *P < 0.05.

When we transfected pcDNA3.1-AKT2 into DLD-1 and HCT-8 cells, which had been stably transfected with miR-650, cell migratory and invasive abilities were again assessed by transwell assays. As expected, miR-650 was able to reduce the migration/invasion rates significantly compared with NC controls when both cell lines were only transfected with pcDNA3.1 plasmids. However, when miR-650 transfectants were transfected with a pcDNA3.1-AKT2 plasmid, migratory abilities of cells were increased back to 210.5 ± 62.9% (*P* < 0.05) in DLD-1 and 166.60 ± 46.1% (*P* = 0.09) in HCT-8, respectively, as compared with that of cells transfected with pcDNA3.1 only (Figure 5A, 5B). The invasive abilities of cells were increased back to 172.86 ± 35.0% (*P* < 0.05) in DLD-1 and 179.6 ± 25.5% (*P* = 0.05) in HCT-8, respectively, compared with controls (Figure 5A, 5C). Futhermore, cell proliferation inhibited by miR-650 could be restored by AKT2 overexpression in DLD-1 cells (*P* < 0.05, Figure 5D).

### Low-expression of miR-650 in CRC cells promotes cancer via AKT2/ GSK3β/ E-cadherin pathway

**Figure 6:**
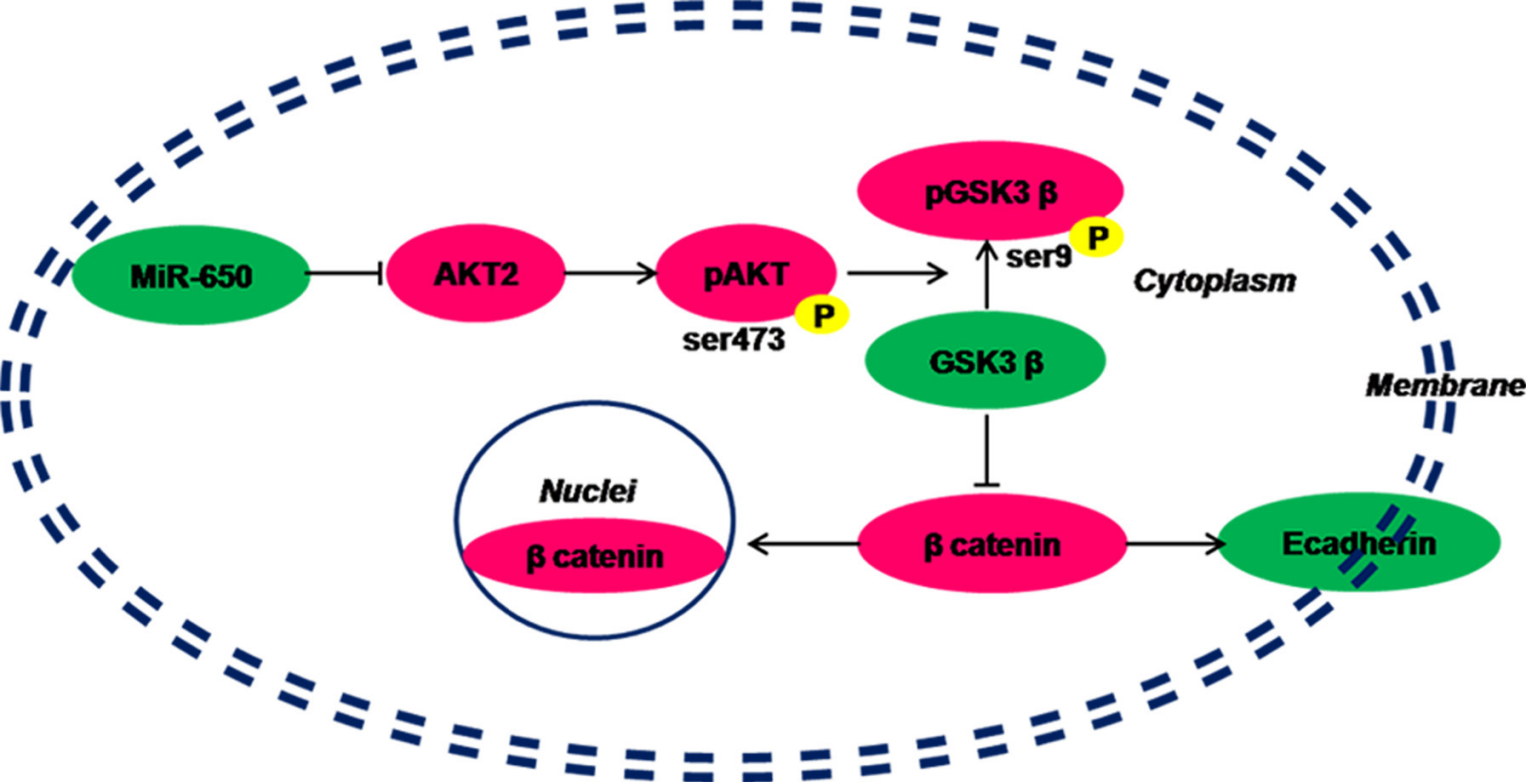
Hypothesis of miR-650 inhibits AKT2/GSK3β/E-cadherin pathway in colorectal cancer cells Green: under-expressed. Magenta: over-expressed.

Our results indicated that miR-650 directly targeted the 3′-UTR of *AKT2*, repressed the expression of AKT2, and then inhibited the activation of the AKT2 pathway. Otherwise, in the relatively lower miR-650 expression CRC tumor cells, the inhibition of AKT2 pathway activation is revived. In this situation, AKT2 and its down streams, such as GSK3β, are over-phosphorylated, which promote the nuclear translocation of β-catenin. The expression of E-cadherin on CRC cell membranes is decreased without the combination of β-catenin. Thus, the cell-cell adhesions between carcinoma cells are weakened and the abilities of migration and invasion of carcinoma cells increase (Figure 6). Therefore, the patients with low expression of miR-650 will have poor prognosis.

## DISCUSSION

Although early-stage (stage I and II) CRC patients have comparatively good prognosis, some of them die of recurrence or metastases after surgical resection. These patients could not be accurately identified by the available pathological/clinical staging or molecular measures. Predictive and prognostic biomarkers are needed to help clinicians to identify the high-risk patients and to treat them accordingly. In addition, those biomarkers also could be potential therapeutic targets.

In this study, we report that miR-650 expression in CRC cells is positively correlated with patient OS in non-metastatic early-stage CRC. Furthermore, miR-650 can identify high-risk patients, even at the same tumor stage (T3 or T4 stage), 18 months later after resection. Although there is no statistical significance at the T2 stage, the similar pattern was still observed. Due to the lack of availability of T1 stage CRCs in this study, such association was not evaluated. Compared with the T2 stage, the tumor at the T3 stage has broken the barrier of muscularis propria and grown into the outermost layer of the colon or rectum. To understand if miR-650 expression is able to predict the progression of T2 or even T1, further validations will be needed.

Our results indicate that miR-650 could be a strong candidate as a prognostic biomarker in early-stage CRC. In addition, we have measured miR-650 expression profiles in epithelial cells derived from snap-frozen colorectal surgical tissues by microarray in a previous study [25]. As shown in Supplementary Table 3, miR-650 expression is significantly decreased in carcinoma compared to normal tissues (*P* < 0.05). These data suggest that miR-650 plays a role as a tumor suppressor gene in CRC progression. Interestingly, miR-650 expression in the left colon is lower than in the rectum and sigmoid in CRC (*P* < 0.05). However, there is no significant difference between left colon and right colon (*P* > 0.05) (Table 1, Supplementary Figure 7). To describe the relationship with miR-650 and CRC location, larger sample power is needed.

We demonstrated that miR-650 could inhibit AKT2 activation and inhibit cell migration and invasion by restoring E-cadherin junctions. AKT2 is overexpressed in metastatic CRC, and suppression of AKT2 could inhibit metastatic ability in CRC cells [28]. We observed that miR-650 could directly bind to the 3′-UTR of *AKT*2 and repress its protein level. This result suggests that AKT2 overexpression in non-metastatic CRC is caused by the loss of miR-650 in CRC cells. With low expression of miR-650 in CRC cells, AKT2 is over-activated, and GSK3β is over-phosphorylated. This promotes the nuclear translocation of β-catenin and the expression of E-cadherin on the cell membrane. This is the reason why epithelial cells with lower expression of miR-650 have fewer cell-cell junctions and are more likely to gain the ability of migration and invasion. Therefore, there is a great possibility that these patients who had lower expression of miR-650 in cancer cells experienced shorter OS, even if they did not have metastasis at the time of first diagnosis.

Furthermore, our data demonstrated that miR-650 would not only be a predictive biomarker but also be a potential drug target. MiR-650 can negatively regulate cell growth, migration and invasion potential of CRC cells *in vitro* and *in vivo*. Over-expression of AKT2 can restore the cell migratory and invasive abilities inhibited by miR-650. The AKT pathway triggers a number of signals in cancer development, making its components attractive drug targets in cancer therapy [29]. For example, the AKT inhibitor MK2206, used in treating metastatic CRC patients, is currently in phase II study [30]. The phase I/II study of Nelfinavir, an AKT signaling inhibitor, in patients with locally advanced rectal cancer has been completed [31]. Our data indicate that the expression of miR-650 is very likely to be a guide of chemotherapy. A non-metastatic CRC patient with relatively higher expression miR-650 will have good prognosis and no need to accept further chemotherapy after surgery. However, a non-metastatic CRC patient with relatively lower expression miR-650 possibly has a bad prognosis and needs further chemotherapy after surgery, e.g. an AKT2 inhibitor.

In conclusion, we have identified that miR-650 has a positive correlation with OS in non-metastatic CRC. MiR-650 could be a potential prognostic biomarker in high-risk non-metastatic CRC patients. Our data also suggest that miR-650 can inhibit CRC progression towards AKT2/GSK3β/E-cadherin pathway, and finally influence patient prognosis. Therefore, miR-650 will likely be a potential therapeutic target in high-risk non-metastatic CRC.

## MATERIALS AND METHODS

### Clinical specimens

The study was approved by the local institutional review boards and written informed consent was obtained from all the 96 patients. The Reporting Recommendation for Tumor Marker (REMARK) guidelines were used to report the study [32]. Routine histological classification according to the WHO Classification of Tumors [33] was used for selection of study samples. All the lesions were diagnosed by two pathologists and independently reviewed by an expert CRC pathologist. Individuals with known inherited syndromes and patients who had received preoperative radiotherapy or chemotherapy were excluded. The prognosis information of patients was taken from Shanghai Municipal Center for Disease Control and Prevention, and telephone follow-ups. The follow-up time ended at the time of the patient's death. Alternatively, follow-up time lasted until the time of this study for good prognosis patients who had survived. Patients who had lived for more than 5 years after surgery were classified as good prognosis, and patients who had died within 5 years from surgery were classified as poor prognosis. All the non-metastasis CRC patients with bad prognosis, who had full survival data from Fudan University Shanghai Cancer Center between 2000–2008, were included in this study. Patients with good prognosis were gender and age matched.

**Figure 7:**
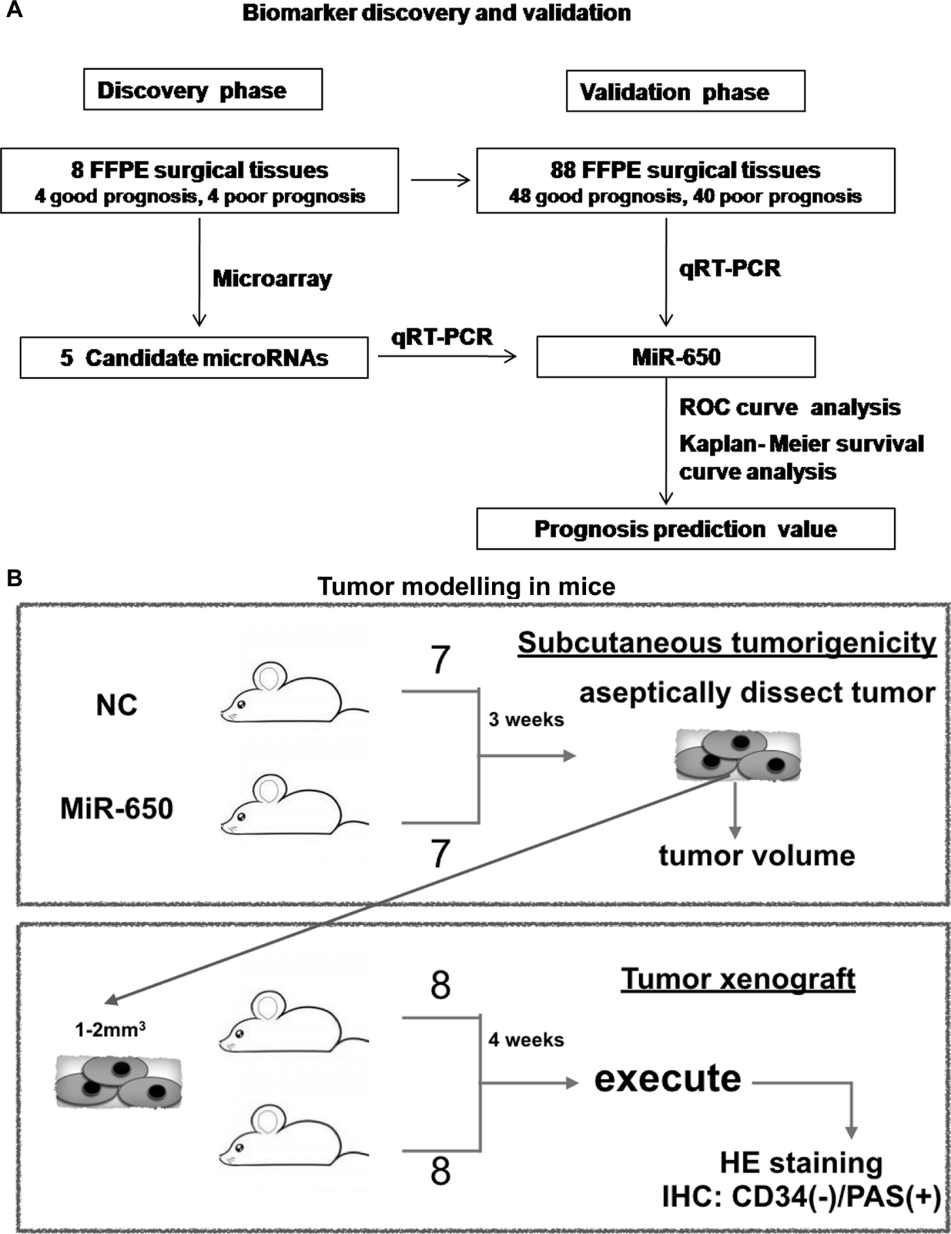
Study design of this study (**A**) Biomarker discovery and validation. (**B**) Tumor modelling in mice.

In the discovery phase, we used 8 FFPE CRC tissues (4 good prognoses and 4 poor prognoses) from Fudan University Shanghai Cancer Center in 2005. Tumor tissues were macrodissected under the light microscope with the control of hematoxylin-eosin (HE) staining slides. We made sure that at least 75 percent of the cells in the sample were cancerous [25]. In the validation phase, we used a total of 96 FFPE surgical tissue samples. Tumor tissues were also macrodissected. Figure 7A described the phases of this study design.

### Microarray hybridization

A total of 8 samples in the discovery phase were sent to Shanghai Biotechnology Corporation, for microarray analysis by Agilent human miRNA (8*15K) V12. Briefly, 100 ng of total RNA was labelled with Cy3. Microarray slides were scanned by XDR Scan (PMT100, PMT5). The labelling and hybridization were performed according to protocols in the Agilent microRNA microarray system. Microarray image information was converted into spot intensity values using Feature Extraction Software 10.7 (Agilent Technologies). Signal data after background subtraction was exported directly into the Gene Spring Software 11.0 (Agilent Technologies) for quantile normalization.

### Total RNA extraction and quantitative RT-PCR

Total RNA was extracted using RecoverAll™ Total Nucleic Acid Isolation Kit (Ambion, Austin, TX). RNA concentrations were quantified by NanoDrop 1000 Spectrophotometer (NanoDrop Technologies, Waltham, MA). MiR-650 expression level was measured by qRT-PCR using Taqman MicroRNA Assays (Applied Biosystems) on a total of 96 FFPE samples (including samples from discovery and validation phases). The expression level of U47 small nuclear RNA was used as an endogenous control. As per MIQE guidelines (PubMed ID 19246619), instead of ‘Ct’, the term ‘Cq’ (for quantification cycle) was used.

### Cell culture, lentiviral transduction and miRNA presursor transfection

Colon cancer cell lines (DLD-1 and HCT-8) and HEK293T were purchased from Shanghai Institute of Cell Biology, Chinese Academy of Sciences (Shanghai, China) and validated by STR profiling by Beijing Microread Genetics Corporation. Cells were grown in RPMI 1640 medium (DLD-1 and HCT-8), DMEM (HEK293T) supplemented with 10% fetal bovine serum. Lentiviral vector was used to transfect miR-650 precursor sequence (miR-650 transfectant) or scrambled control sequence (negative control) to DLD-1 and HCT-8 cells. HCT-8 and DLD-1 cells were transfected with pre-miR-650 and paralleled negative control (Life technologies, Carlsbad, CA) respectively using Lipofectamine 2000 transfection reagent (Life technologies, Carlsbad, CA).

### Cell proliferation, migration and invasion assay

DLD-1 and HCT-8 cells (about 7000 cells per well) of miR-650 transfectants or negative controls (NC) were seeded in 96-well plates. Cell number was measured by Cell Counting Kit-8 (Dojindo) on different times (0, 24, 48, 72, 96 h). Cell migration and invasion ability were performed using transwell insert (8 μm, Corning). Cells were added to the upper chamber in RPMI 1640 medium supplemented with 1% fetal bovine serum. Medium containing 20% serum was placed in the lower chamber. For the invasion assay, inserts were pre-coated with extracellular matrigel (BD Biosciences). Penetrated cells were stained with 0.1% crystal violet and counted.

### Tumor mice model

The animal experiments were approved by our institutional ethical board and reported according to the ARRIVE guidelines [34]. Nude BALB/c mice (SLRC Laboratory Animal) weighing approximate 20 g, male, 6–8 weeks of age, were used. Animals were maintained in a laminar flow cabinet under specific-pathogen-free (SPF) conditions, kept at 26–28°C, humidity 40–60%. 3–4 mice were housed in one cage, and were fed twice per day, with the cycle of 10 hours of day light and a 14 hour night. Figure 7B described the study design. Firstly, 4 × 10^6^ DLD-1 cells of miR-650 transfectants or NC controls were subcutaneously (s.c.) injected into nude BALB/c mice (7 mice per group). Tumor size was measured at regular intervals twice per week, and the tumor volume was calculated using the formula: volume = 1/2 × (length × width^2^). After three weeks, tumors were aseptically dissected. In the next phase, to conduct a xenograft mice model, 16 nude BALB/c mice were divided into two groups (8 mice per group). Mice were anesthetized and sections of tumor tissues (1–2 mm^3^) of subcutaneous tumor model were planted on the serosa of the ileocecus. After surgery, the mice were injected subcutaneously with penicillin to prevent infection, once a day, for 3 days. The tumor xenograft mice were sacrificed four weeks after the surgery (one mouse died of intestinal obstruction three weeks after the surgery in the NC group). Tumor tissues were used for HE staining and immunohistological staining (IHC) for CD34 (2150–1, 1:200, Epitomics), Ki-67 (16667, 1:100, Abcam). Vasculogenic mimicry (VM) in 14 tissue samples derived from the mouse xenograft model were identified by CD34/PAS (periodic acid-schiff) dual-staining. The channels which enclosed by tumor cells and PAS (+) / CD34 (−) were defined as VM [35].

### Target prediction

Targets of miR-650 were predicted by miRecords (http://c1.accurascience.com/miRecords/). To increase the accuracy of prediction, the genes predicted by at least 4 of 11 databases (DIANAmicroT, Microinspector, miRanda, MirTarget2, miTarget, NBmiRTar, PicTar, PITA, RNA22, RNAhybrid and TargetScan) were selected as candidate targets. The KEGG database (http://www.kegg.jp/kegg/download/kegtools.html) was used to map the predicted targets to pathways.

### Cloning and construction of AKT2 and related plasmids

The three nucleotides harboring four seed sequences in the 3′-UTR of *AKT2* or two mutated seed nucleotides (Supplementary Table 4) were generated by Jieli Biology (Shanghai, China). They were cloned into pmirGLO vector. Full length cDNA of *AKT2* was generated from HEK293T cells, and cloned into pcDNA3.1 vector. All the resulting constructs were confirmed by DNA sequencing.

### Luciferase activity assay

HEK293T cells were cotransfected with 40 nM miR-650 precursor or negative control (Ambion) and 50 ng pmirGLO (Promega) constructs harboring the seed sequences of 3′-UTR of *AKT*2. The seed sequences were mutated in mutated reporter constructs. Cells were collected 48 hours after transfection and analyzed with a Dual-Luciferase Reporter Assay (Promega). Firefly luciferase activity was normalized to Renilla luciferase activity accordingly. ING4 was reported to be a miR-650 target [22] and was selected as a positive control.

### Western blot analysis

Total protein and nuclear protein were harvested 48 hours after transfection (NE-PER Nuclear and Cytoplasmic Extraction Reagents). The protein expression of following antibodies were detected: AKT2 (sc-5270, 1:500, Santa Cruz Biotechnology), p-AKT (Ser473, 1:1000, no. 9271, Cell Signaling Technology), GSK3β (sc-9166, 1:1000, Santa Cruz Biotechnology), p-GSK3β (Ser9, 1:1000, no. 9336, Cell Signaling Technology), E-cadherin (24E10, 1:1000, no. 3195, Cell Signaling Technology), and β-catenin (D10A8, 1:1000, no. 8480, Cell Signaling Technology). The expression level of GAPDH (sc-166574, 1:1000, Santa Cruz Biotechnology) and TBP (2H3B2, 1:5000, protein tech) were used as endogenous controls.

### Immunofluorescence

1 × 10^4^ DLD-1 and HCT-8 cells of miR-650 transfectants or NC controls were seeded into 24-well plate. After being blocked with 10% goat serum for half an hour, cells were incubated with anti-E-cadherin antibody (1:400, Abcam) for 1 hour. Followed by incubation with Cy3-conjugated secondary antibody (1:500, proteintech) for 1 hour, cells were stained by DAPI and observed under confocal microscope (Leica, Microsystems).

### Statistical analysis

Unpaired *t*-test with Benjaminie-Hochberg correction was used for differentially expressed microRNAs in 8 samples in discovery phase. Receiver operating characteristic (ROC) curve analysis [36] was performed to determine the specificity and sensitivity of miR-650 as predicted biomarker in a total of 96 samples in validation phase. The area under curve (AUC) and corresponding *P* values from a Wilcoxon signed rank test were performed further. Kaplan-Meier survival curve analysis was performed to observe survival rate of miR-650. Associations between gene expression and OS were quantified by HRs and corresponding 95% CIs from Cox proportional hazards regression. Data are shown as mean ± standard deviation. Differences between groups were determined by a two-tailed *t*-test. Statistical analysis was performed using MedCalc software.

## SUPPLEMENTARY MATERIALS TABLES AND FIGURES



## Abbreviations

CRC: Colorectal cancer
miRNA: microRNA
OS: overall survival
FFPE: formalin-fixed, paraffin-embedded
qRT-PCR: quantitative reverse-transcriptase-polymerase-chain-reaction
NC: negative control
ROC: Receiver operating characteristic
AUC: area under curve
CD34: cluster of differentiation 34
PAS: periodic acid-schiff
TNM: T, primary tumor; N, regional lymph node involvement; M, metastases
