# Supplementary material for: MiR-650 represses high-risk non-metastatic colorectal cancer progression via inhibition of AKT2/GSK3β/E-cadherin pathway
Source: Oncotarget. 2017 May 10;8(30):49534–47. doi: 10.18632/oncotarget.17743 (PMC5564786; doi:10.18632/oncotarget.17743)
Supplement: Supplementary file 1 [file oncotarget-08-49534-s001.pdf]

MiR-650 represses high-risk non-metastatic colorectal cancer progression via inhibition of AKT2/GSK3β/E-cadherin pathway

Supplementary Materials

Supplementary Table 1: Candidate miRNA biomarkers in CRC tissues among patients with different prognosis by miRNA microarray

microRNA	*P.* Value	Fold Change (Poor/Good)
hsa-miR-650	0.050	0.3
hsa-miR-146a	0.027	0.4
hsa-miR-365	0.030	0.5
hsa-miR-125a-5p	0.029	2.6
hsa-let-7e	0.033	2.7

Supplementary Table 2: The potential targets of miR-650

<table>
<tr><td>Position 174-181 of AKT2 3′ UTR

hsa-miR-650</td><td>5′...GGCCCCUGCAGCCCCUGCCUCCA...
| | | | | | |
3′ CAGGACUCUCGCGACGGAGGA</td></tr>
<tr><td>Position 182-188 of AKT2 3′ UTR

hsa-miR-650</td><td>5′...CAGCCCCUGCCUCCAGCCUCCAG...
| | | | | | |
3′ CAGGACUCUCGCGACGGAGGA</td></tr>
<tr><td>Position 358-364 of AKT2 3′ UTR

hsa-miR-650</td><td>5′...UGUCGUGCUGGUGUCUGCCUCCG...
| | | | | | |
3′ CAGGACUCUCGCGACGGAGGA</td></tr>
<tr><td>Position 732-738 of AKT2 3′ UTR

hsa-miR-650</td><td>5′ ... GGGGCCAAACCUGCCUGCCUCCC...
| | | | | | |
3′ CAGGACUCUCGCGACGGAGGA</td></tr>
</table>

Supplementary Table 3: Expression of miR-650 in colorectal epithelial cells on microarray

	Fold (vs Normal (n = 11))	*P.*Value (vs Normal (n = 11))
Adenoma (n = 23)	0.60	0.038
Dukes' A (n = 14)	0.18	0.000
Dukes' B (n = 20)	0.14	0.000
Dukes' C (n = 23)	0.19	0.000
Dukes' D (n = 10)	0.30	0.022

Supplementary Table 4: The seed nucleotides and mutated nucleotides of AKT2

1st, 2nd Seed Wild	5′-CCAGCGGCCCCTGCAGCCCCTGCCTCCAGCCTCCAGCCTCACCTTTGTGCCCAGT-3′
1st Seed Mutant	5′-CCAGCGGCCCCTGCAGCCCCTTCATCTAGCCTCCAGCCTCACCTTTGTGCCCAGT-3′
2nd Seed Mutant	5′-CCAGCGGCCCCTGCAGCCCCTGCCTCCAATTTCTAGCCTCACCTTTGTGCCCAGT-3′
3rd Seed Wild	5′-CCTGGGTGTCGTGCTGGTGTCTGCCTCCGCGCTGCTGCATCTGGACGAAT-3′
4th Seed Wild	5′-CACATTTGGGGCCAAACCTGCCTGCCTCCCAGCCCCGTGCCTTACTAGT-3′

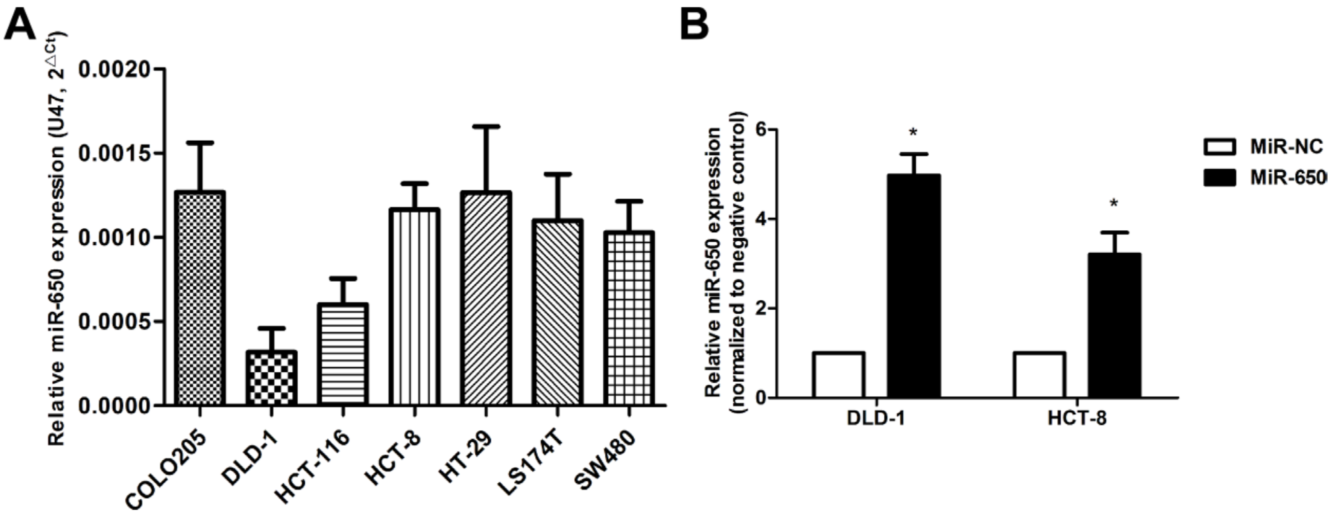

Supplementary Figure 1: Expression levels of miR-650 in colon cancer cells. (**A**) The basal expression levels in seven colon cancer cell lines. (**B**) Expression levels of miR-650 in DLD-1 and HCT-8 transfectants. MiR-650 precursor was transduced to DLD-1 and HCT-8 cells by lentiviral vectors. * $P < 0.05$.

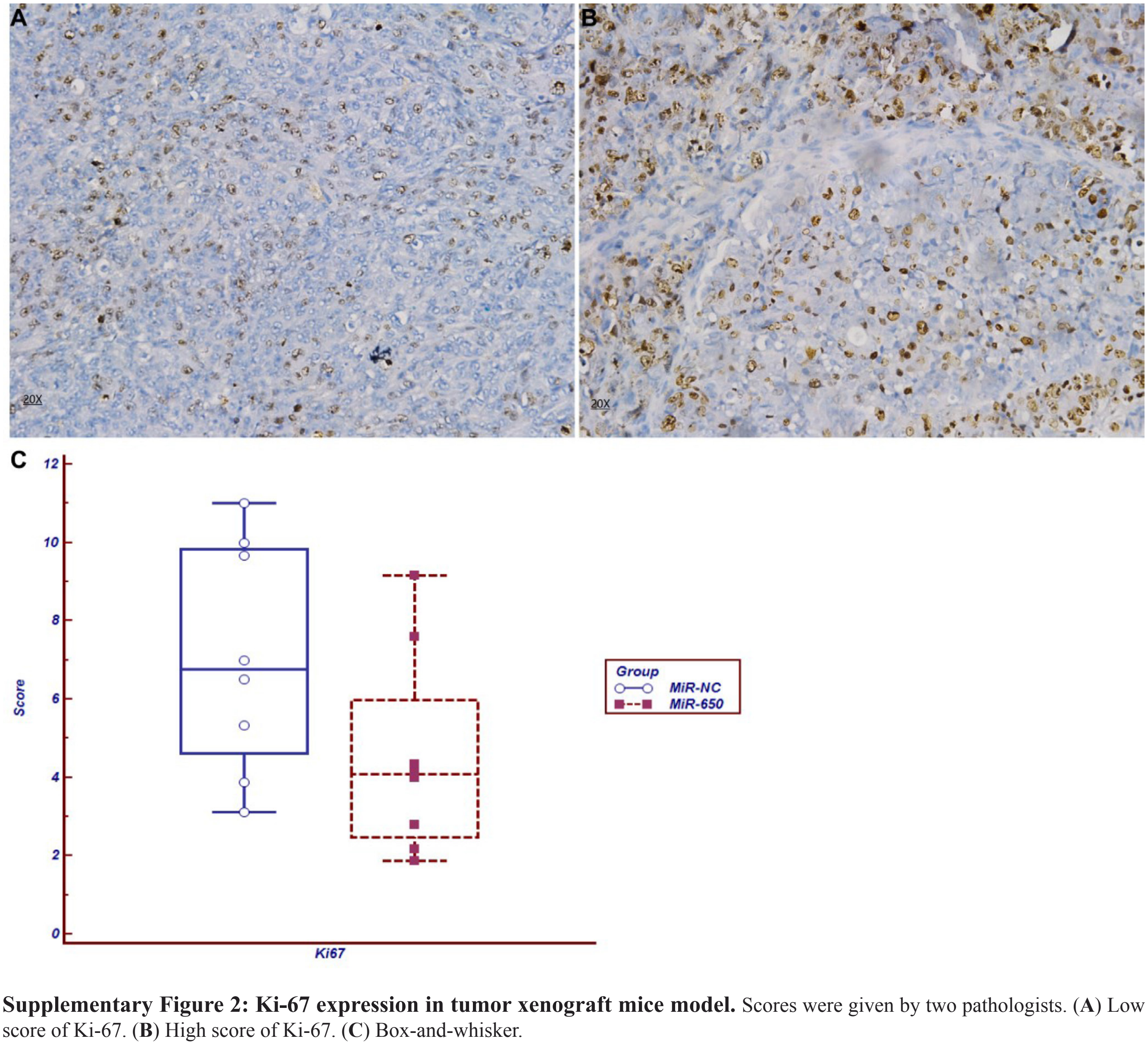

Supplementary Figure 2: Ki-67 expression in tumor xenograft mice model. Scores were given by two pathologists. (**A**) Low score of Ki-67. (**B**) High score of Ki-67. (**C**) Box-and-whisker.

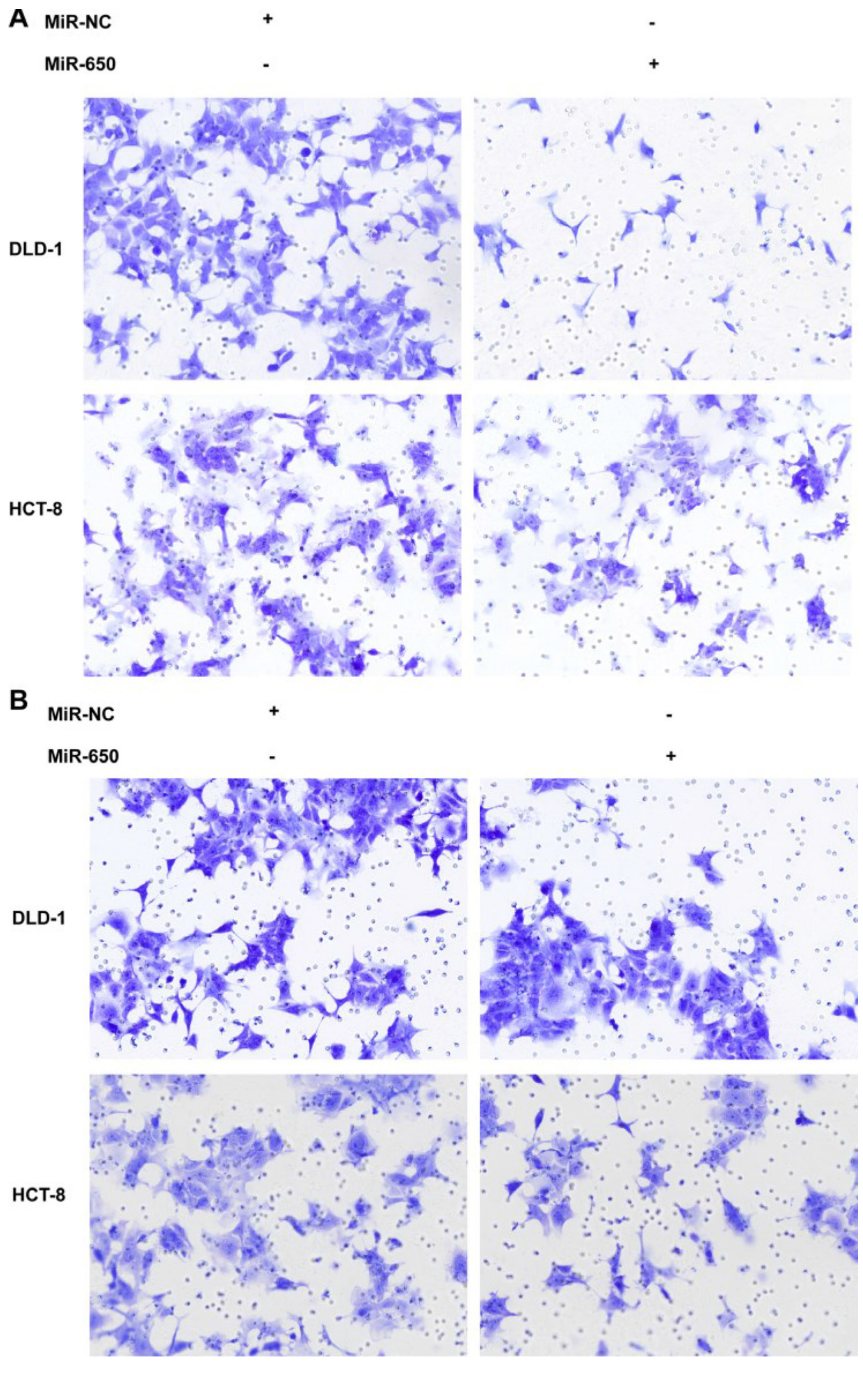

Supplementary Figure 3: Effects of miR-650 on regulating migratory and invasive abilities in DLD-1 and HCT-8 cells (200×). (**A**) Cell migratory abilities. (**B**) Cell invasive abilities.

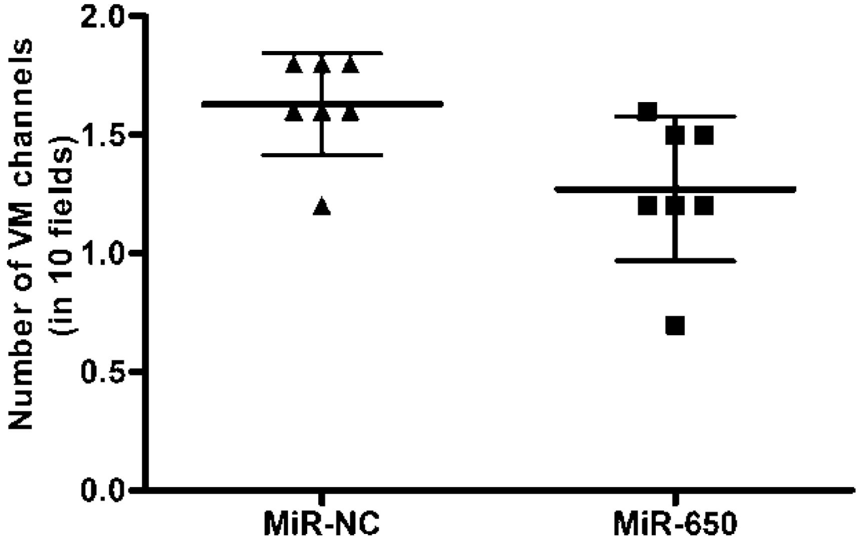

Supplementary Figure 4: Number of VM channels in xenograft mice model.

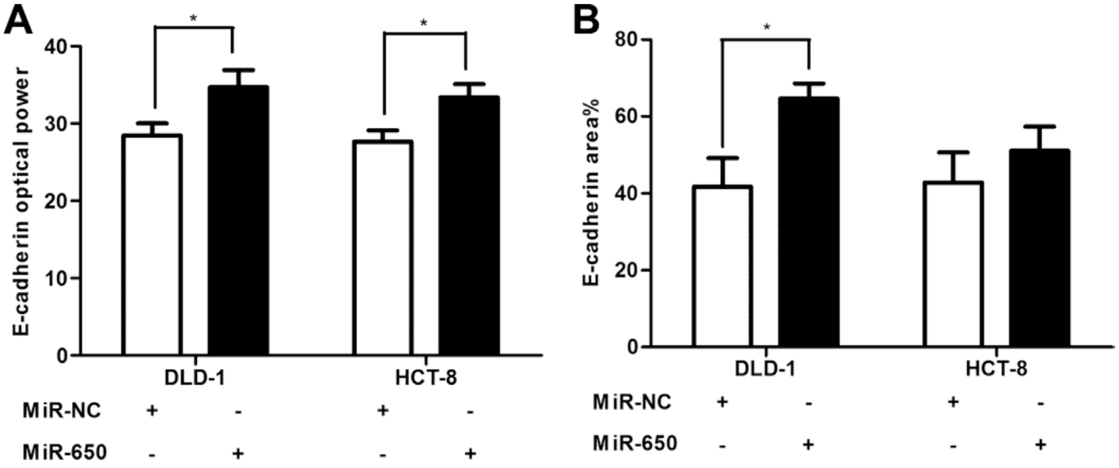

Supplementary Figure 5: Expression of E-cadherin in DLD-1 and HCT-8 cells. (**A**) E-cadherin optical power on cell surface.*P = 0.03 in DLD-1 and *P = 0.02 in HCT-8. (**B**) E-cadherin area% on cell surface.*P = 0.01.

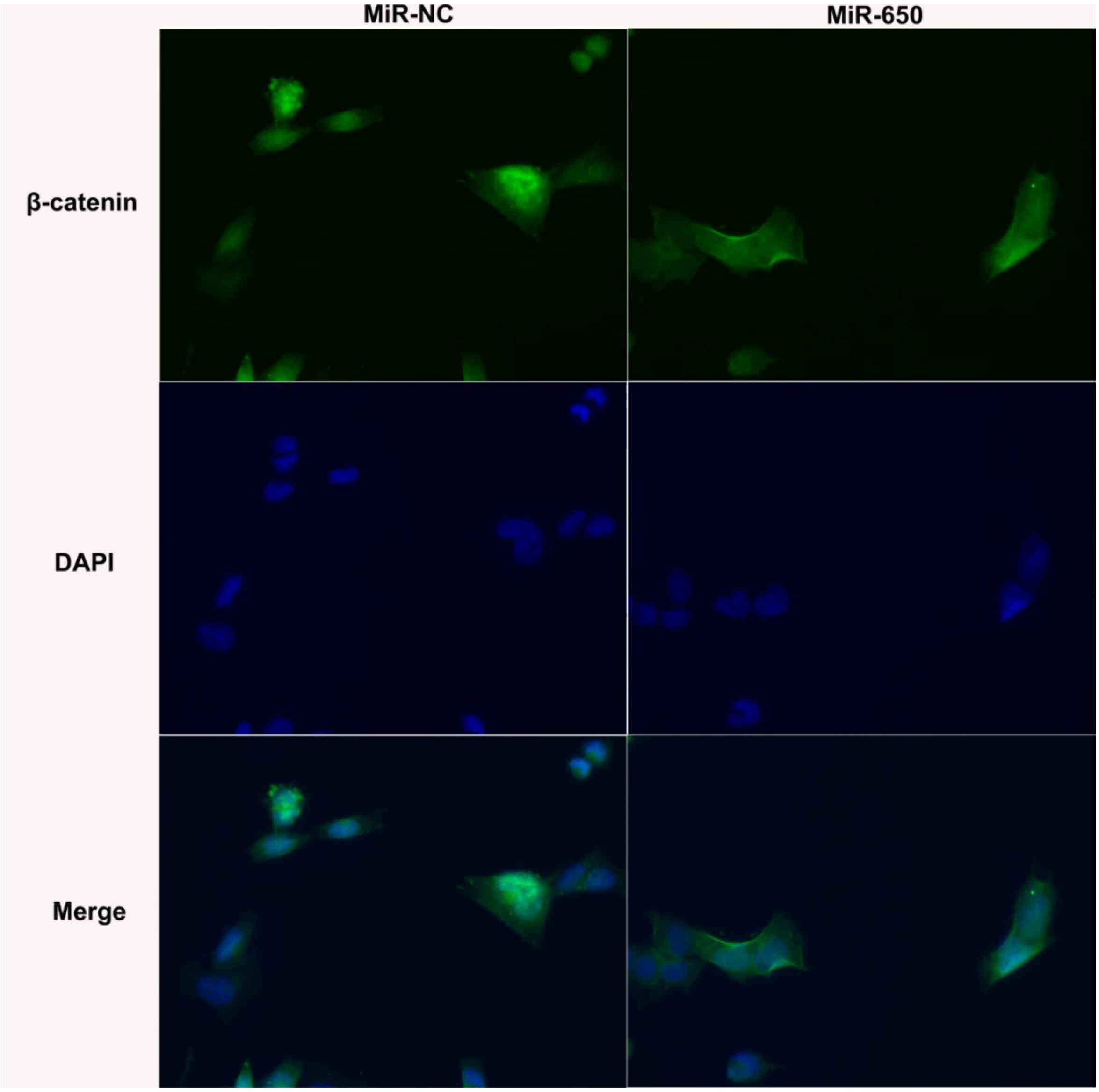

Supplementary Figure 6: Expression of β-catenin in DLD-1 cells.

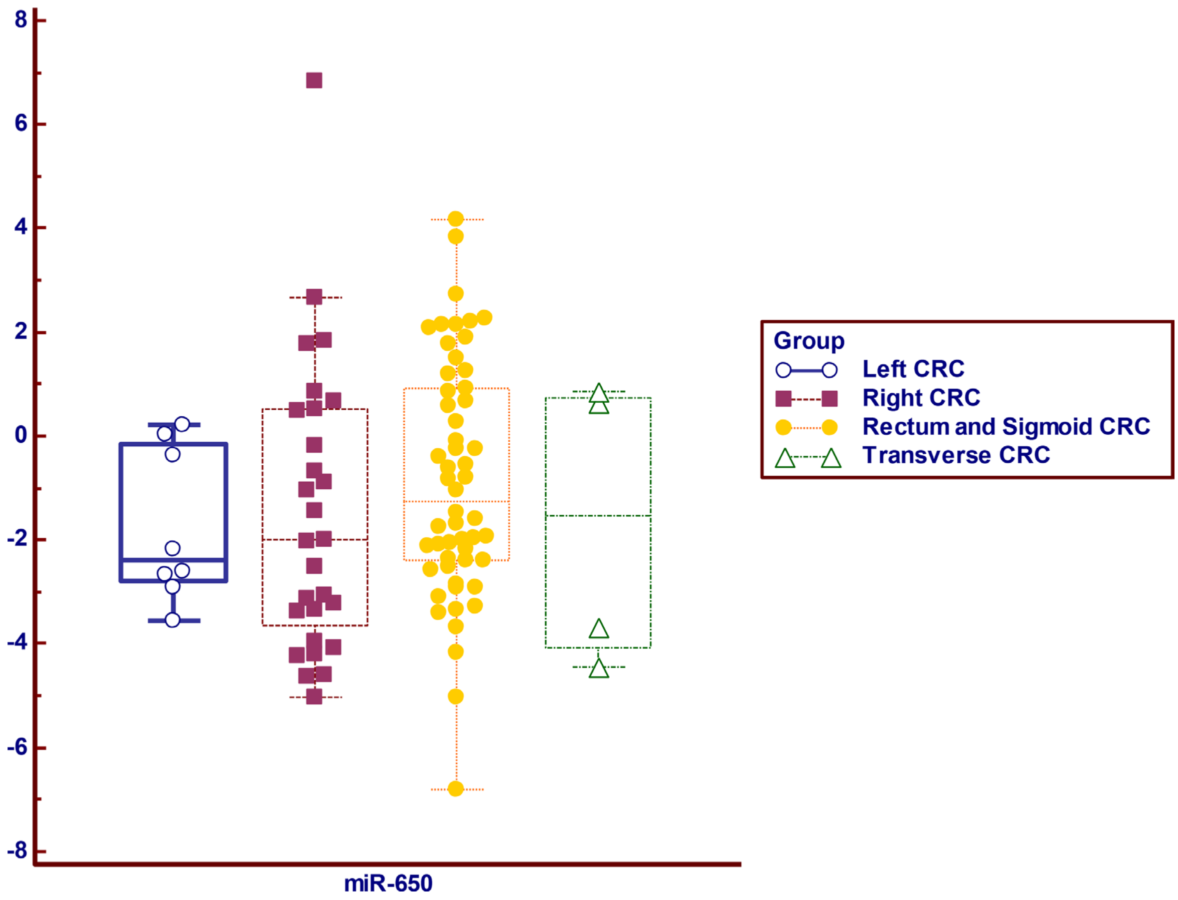

Supplementary Figure 7: MiR-650 expression in different location of CRC.
